# Rethinking Competence: A Nexus of Educational Models in the Context of Lifelong Learning

**DOI:** 10.3390/pharmacy8020081

**Published:** 2020-05-08

**Authors:** Dalia Bajis, Betty Chaar, Rebekah Moles

**Affiliations:** School of Pharmacy, Faculty of Medicine and Health, University of Sydney, Sydney, NSW 2006, Australia; betty.chaar@sydney.edu.au (B.C.); rebekah.moles@sydney.edu.au (R.M.)

**Keywords:** competency, competency assessment, education models, lifelong learning, pharmacy education, professional development

## Abstract

Competency-based education (CBE) “derives a curriculum from an analysis of a prospective or actual role in modern society and attempts to certify students’ progress on the basis of demonstrated performance in some or all aspects of that role”. This paper summarizes pertinent aspects of existing CBE models in health professions education; pharmacy education presented as an example. It presents a synthesis of these models to propose a new diagrammatic representation. A conceptual model for competency-based health professions education with a focus on learning and assessment is discussed. It is argued that various elements of CBE converge to holistically portray competency-based learning and assessment as essential in initial education and relevant to practitioners’ continuing professional development, especially in the context and importance of pursing lifelong learning practices.

## 1. Background

In medical education, competency-based education (CBE) is defined as “education that derives a curriculum from an analysis of a prospective or actual role in modern society and attempts to certify students’ progress on the basis of demonstrated performance in some or all aspects of that role” [1]. We suggest that the same definition can be used for training other health care professionals, including pharmacists. Competencies (singular: competency) can simply be viewed as building blocks of competence. They are observable components of knowledge, skills, attitudes and values expressed as actual behaviour which can be measured and assessed [2]. However, in health professions education, understandings of what pertain to competency have evolved over time, leading to a variety of interpretations with contested “meanings” [3]. Competence (the ability to carry out a job or task) is also considered multi-faceted, context-driven and dynamic, but generally prescribes the “integration of abilities across multiple domains and aspects of health care professional performance in a certain context” [4].

Apart from CBE being a resurgent paradigm in initial education (undergraduate and professional degrees) for curricular development and assessment, competency-based training programs and competency frameworks have been advocated for professional development skills and advanced or specialised practice [5,6]. The very definition of continuing professional development (CPD) described by Alsop “the process of the on-going education and development of health care professionals, from initial qualifying education and for the duration of professional life, in order to maintain competence to practice and increase professional proficiency and expertise” (cited from [7], (p. 1)) emphasizes that competence needs to continue to be developed as well as maintained. But competence, as we will describe later, is a “moving target”; and as long as clinical, social, technological and ethical situations continue to evolve so will competence and the competencies associated with it. This is why it is imperative to highlight the importance of engaging in lifelong learning as a grounding practice that will guide learners (students or practicing professionals) to navigate the ever-changing world, and not just to maintain competence. Lifelong learning is no longer viewed as a domain only relevant for adult learning or continuing education, but increasingly a practice that needs to be instilled in students early in their learning. Moreover, lifelong learning embraces improvement of knowledge, skill and personal competence in order to participate actively within society across the lifespan and not just in working life (cited from [8], (p. vii)).

Although CBE is touted in health professions education, its implications for clinical practice (particularly in terms of performance in work practice and patient outcomes) remain challenged by critics [4]. One such criticism of CBE is that it works on the premise of adequacy rather than excellence, or that it applies an atomistic approach, breaking down complex skills into their constituent parts to facilitate assessment [9]. In doing so, the complexity and context of real professional practice is often neglected, making it an insufficient measure of professional capacity. McLellan et al. assert: “true competence is a socially situated concept which demands that learners are able to adapt to uncertainty, respond to the different contextual features of different workplaces … (and) from a cognitive psychology perspective, achieving true competence demands expertise” [9]. Competency-based education, with its various (and changing) definitions and dimensions, still holds great promise for health professions’ training worldwide and across different levels of education, scopes of practice and professional development. Therefore, what follows is a brief but holistic summarization of existing CBE educational models that the authors suggest applies to initial education and potentially to post-registration training or CPD.

## 2. Nexus of Educational Models

**Figure 1:**
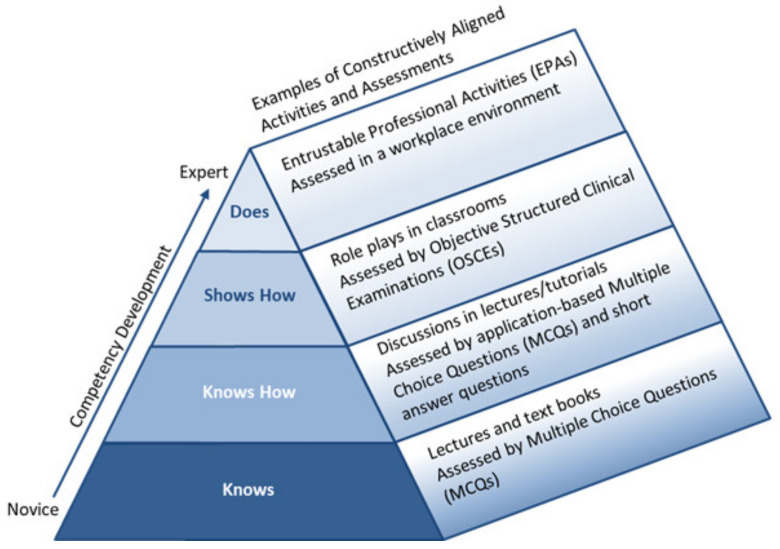
Constructively aligned tent of competency assessment. Adapted from: Miller’s prism of clinical competence [10], Biggs’ constructive alignment [11], and Dreyfus & Dreyfus’ development of expertise model [12].

In Section 3, education models will be described and have been adapted as shown in Figure 1.

In CBE, it may be desirable to establish and specify competency levels for evaluating student achievement [13]. A widely quoted model in health professions education is Miller’s (1990) prism of clinical competence [10]. Miller created a construct, first presented for medical trainee education, as an approach to rank and assess the clinical competence of learners, emphasising that CBE and assessment acknowledge the assimilation of knowledge into performance [10]. As a framework, Miller’s construct distinguishes between knowledge at the lower levels and action in the higher levels of an educational experience. It argues that, to truly know whether learners are achieving what they are supposed to achieve, they should be assessed in the setting that they are expected to practice in. At the base of Miller’s prism, knowledge acquisition by students or qualified health professionals is the knowledge required in order to carry out particular professional tasks. *Knowing how* to use and apply this knowledge is achieved by utilising the skills necessary to acquire, analyse and interpret supplementary information to translate into a rational diagnostic or management plan (for medical trainees). Performance and action represent the higher order objectives in Miller’s framework, posing real challenges in terms of assessment and evaluation of learners or qualified professionals on their ability to demonstrate that they can *show how* and actually *do* what is required of them in clinical practice.

Recently, Miller’s prism had a third dimension added, to incorporate the domains of Bloom’s taxonomy to highlight how and when learners are to be assessed for competence [14]. Methods used for teaching, learning and assessment can be guided by these constructs to facilitate the developmental progression towards competence. As learners acquire knowledge and skills, appropriate assessment methods should be constructively aligned to the competency being tested [11,14]. Assessments that test fact-gathering and interpretation of knowledge target the *knows* and *knows how* level of knowledge acquisition, whilst demonstration of learning and integration of performance into practice target the *shows* and *does* levels of Millers’ prism.

Rather than aiming for adequacy, focusing on developing expertise in students is an area that has interested educators and researchers in pharmacy in recent years [15]. That is, aiming for expertise, not just competence. Going beyond competence was previously examined by the Dreyfus development of expertise model [12], which proposes that expertise develops over several stages: novice, advanced beginner, competent, proficient and expert [15]. Several characteristics differentiate between experts and novices. Experts have built a substantial knowledge foundation which affects what they notice, and how they organise, represent and interpret data, using their clinical and professional judgement [15]. At the other end of the scale, novice learners follow rules, spend time memorising facts, and have no discretionary judgement [13].

To illustrate this type of education model, a study by Bajis et al. presented a learning activity conducted with final year pharmacy students, aimed at teaching students the required skills to carry out medication history-taking [16]. Students in this study had not learned to perform medication reconciliation in previous training and had limited interactive learning opportunities. According to the Dreyfus model, students appeared to demonstrate characteristics of ‘novice’ and ‘advanced beginner’ stages simultaneously. Advanced beginner, in this context, means that they were able to provide partial solutions to unfamiliar situations, but unable to see the bigger picture [13]. The assessment, criteria-based on a set of competencies, initially challenged students, but with further supportive coaching and feedback, which encouraged self-reflection on performance, it appeared possible to enhance the instruction stage to a more ‘competent’ level of learning. In this example, competent performance was the ability of students to identify medication discrepancies and prevent harm to the “patient”. Whilst there is no clear method to stage learners, certain behaviours, such as those described above, might be helpful to instructors to tailor individual learner needs to help advance their skills to the expert level.

While the Dreyfus model describes characteristics of the developmental stages to expertise, the central tenets of mastery learning, as conceptualised by Bloom, are based on the idea that educational excellence is expected and can be achieved by all learners and little or no variation in measured outcomes will be seen among learners [17]. Mastery learning has been described as an “especially stringent” form of CBE, emphasising acquisition of competencies measured rigorously against predetermined achievement standards, without limiting the time needed to reach the outcome [18]. Key features of this approach include: baseline testing, clear learning objectives, engagement in educational activities, a set minimum passing standard, formative assessment, advancement to the next level occurs when mastery is achieved, and continued practice or study on an educational level until the mastery standard is reached [17]. Educators in the field assert that the mastery approach to learning is important for the acquisition of critical thinking and problem-solving skills, and for promoting lifelong learning attributes that many schools of pharmacy today desire in their graduates [19].

## 3. A New Theoretical Model

**Figure 2:**
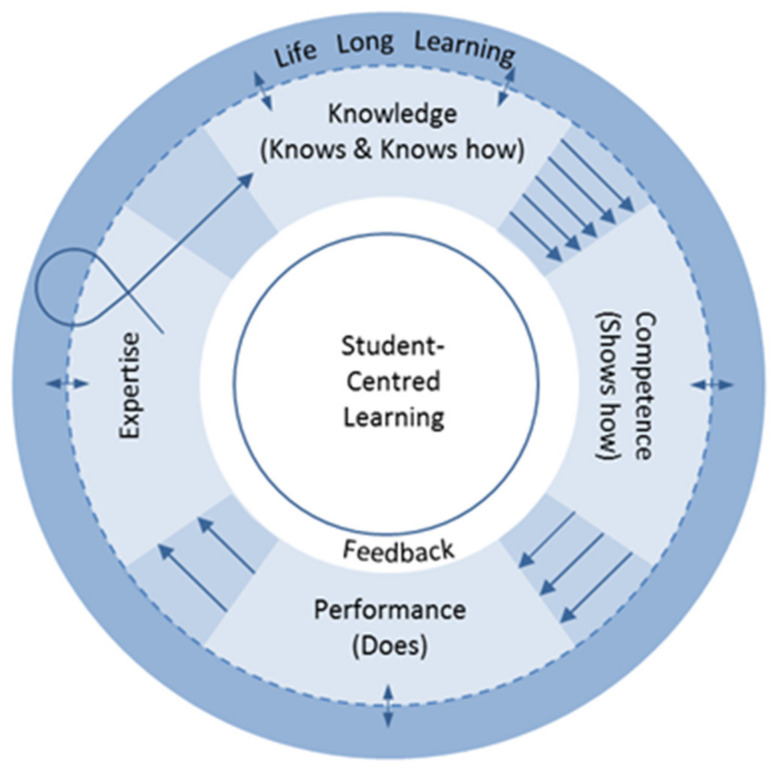
Competency-based learning and assessment cycle [20].

To that end, in the context of the aforementioned educational models, D Bajis proposed a conceptual model for competency-based learning and assessment, towards expertise in undergraduate pharmacy education [20], presented in Figure 2. The model was built from the foundations of Miller’s assessment of clinical competence [10], Dreyfus’s model of developmental stages of expertise [12], and mastery learning [17]. The broad objective of this proposition was assessing students as they move from knowledge base domains to acquirement of expertise.

The diagram in Figure 2 illustrates a model within which learning, and assessment, might occur. The model emphasises that knowledge, competence, and performance toward the acquisition of expertise, need to be integrated domains and are involved in a continuous loop of updates from practice and research. It is important to acknowledge that no single assessment method or model can possibly capture all the data required for judgement of the delivery of professional services by a pharmacist or pharmacy student. The model also assumes that skills acquired in one domain may indeed be transferred from one domain to another, allowing students to acquire knowledge perhaps more quickly as they rotate through related cycles.

The first feature of this model is where the *knows* and *knows how* of Miller’s prism are combined and correspond to the “test cognition” or knowledge domain, similarly addressed by previous researchers [13]. Secondly, and based on definitions of competence, this model highlights the ability to do something adequately or efficiently [21], emphasising the action part of a task. In contrast to Miller, competence in this model corresponds to *shows how* as a construct of competence. *Shows how* refers to assessment of a learner’s performance using artificial simulation exercises, such as objective structured clinical examinations (OSCEs) and therefore under supervision [13]. Performance denoting action, the third domain in this cycle, links with a learner’s clinical practice in the workplace environment (with minimal supervision) [13]. The third feature in this model, is the representation of expertise as the ultimate developmental goal in the learning experience. Professional expertise has many contextual dimensions that affects its development (e.g., social context, self-regulation, deliberate practice), but overall it refers to an individual who has rich experience and takes actions automatically, using intuitive judgement in solving problems [13].

Another feature of this model, and perhaps the most significant, is the circular depiction of transference of knowledge into application. The dotted circular line depicts the constant exchange of new knowledge from experience, practice and research, therefore involving learners in lifelong learning activities [22]. In contrast to Miller’s prism or the modified Dreyfus model which use a pyramidal representation of a hierarchal development of expertise, this model presents development of professional competence and expertise embodied in a continuous cycle of lifelong learning commitment. This accounts for competencies needed for self-directed learning, which are best developed in learners before entering practice [23]. Such expectations are disseminated in recent accreditation standards and associated guidance for professional pharmacy degree programs [23,24]. The essence of maintaining expertise in performance in the workplace is illustrated by a looped-arrow re-entering the learning and assessment cycle; a depiction of constant exposure to various workplace contexts in practice and the need for self-reflection on continuing education needs.

It is crucial that at the core of competency-oriented education is the concept of student-centred learning—depicted in the model by the central white circle. The student-centred learning approach shifts the focus from the teacher to the learner and fosters motivation and incentive to learn [25]. Implications of this approach for assessment include learner involvement in how they need to be evaluated and demonstrate their learning [25]. This approach should also be coupled with effective formative assessment, feedback and observation mechanisms—depicted in the model, which are central to competency-based assessment.

Today, one must recognise more than ever before that education needs to do more than give students opportunities to acquire knowledge and skills, but that it is a solid pedestal for social and personal development, and well-being. So what if student-centred *learning* in Figure 2 is substituted with person-centred learning? A progression on this proposed model which would allow educators to seriously pay special attention to the “person” in the learning journey, is to frame it in such a way. Here, the field of positive psychology adds yet another dimension to this proposition, one that explores how to nurture the student’s educational journey to one of profound personal-development and growth. Personal development, also known as self-development/self-growth, is a lifelong process encapsulated in formal and informal activities for developing not only students’ competence, but their self-awareness and self-reflection. From Aristotle, who provides a view on personal development as “human flourishing” or “living well”, to Martin Seligman who asserts heightened self-awareness and a focus on an ultimate goal, essential components of personal development in education must be endorsed [26]. For example, positive education, a concept founded by Seligman in which he incorporates positive psychology into education models, is premised on students’ positive emotions, engagement, relationships, meaning and accomplishment for long-term happiness and well-being [27]. It is proposed that educators ought to explore such approaches to improve students’ well-being and create opportunities for personal fulfilment emphasising the process of learning and focus on strengths. In alignment with this added layer to enhance students’ competency-based learning experience, an alternative modus operandi to student-centred learning becomes person-centred learning.

In rethinking the models presented in this paper so far, we have invited educators to reconsider theory-to-practice gaps in pharmacy education, gaps that could be consequential of possibly insufficient integration of all the necessary aspects of expertise acquirement during the training of pharmacy students. Based on this synthesis, we suggest that educators consider evaluating their learning and assessment philosophies and practice models based on a model or framework of expertise and personal development such as the one proposed in Figure 2.

## 4. Competence—A Moving Target

Preparing graduate pharmacists with a predetermined standard of competence in isolated aspects of the skills needed for practice is unlikely to make new graduates become effective, safe and professional pharmacists, because the tasks, the contexts in which they have to be applied and the interaction between the two are very complex [28]. It is necessary to acknowledge that the environment in which performance of skills takes place—what some refer to as the social context [9], is multifaceted. The interplay between students’ cognitive processing of their knowledge, skills and attitudes, and the environment of workplace demands cannot be overstated. Students may work in teams of healthcare professionals in diverse health systems, and every time a new context arises, their learning must be transferred to practice [29]. This environment is dynamic, characterised by regulatory changes, business priorities, workforce changes, information systems and clinical initiatives [30].

Added to knowledge-based competence, for example, the importance of emotional intelligence, (a type of social intelligence) consisting of several personal and social competencies (self-regulation, motivation, self-awareness, social skill, and empathy) is becoming increasingly apparent for effective teamwork and leadership within the workplace [31]. Factoring these important competencies into curricula means that schools of pharmacy need to embrace evidence-based guidelines to develop, teach and asses these socially-oriented skills and attitudes to pharmacy students. Ideally, and as Holmboe et al. (2010) summarise, “assessment facilitates the developmental progression of competence” [32]. However, to implement and improve a CBE assessment system, assessment should be viewed in the context of a complex and adaptive system [32], one that is characterised by evolving competencies, multiple “agents” (e.g., faculty members, peers, patients, other health care professionals), and multiple assessment tools (e.g., feedback, simulation), in collaboration with the learner in a CBE training model [32].

Competence manifests as a moving target; evolving rapidly, leaving academic pharmacy with the task to project future practice and develop needs-based curricula [33]. Jungnickel et al., in 2009, described professionalism, self-directed learning, leadership and advocacy, inter-professional collaboration and cultural competency as “cross-cutting” competencies essential for pharmacy curricula design [33]. In 2017, McLaughlin et al. identified eight overarching competency themes considered essential for success of pharmacists in today’s rapidly evolving health care environment [28]. These were: critical thinking and problem solving, collaboration across networks and leading by influence, agility and adaptability, initiative and entrepreneurialism, effective oral and written communication, accessing and analysing information, curiosity and imagination, and self-awareness [28]. These are only two studies of many representing steps in understanding how to best prepare pharmacy students for the emerging health care needs of society [28].

As long as pharmacy education remains challenged by shifting health system contexts, pharmacists must remain skilful and vital members of the health care team in all clinical settings, practicing at their fullest extent of licensure and education [34]. In professional pharmacy education, for some, although “the outcome goal of a particular educational stage may not be for learners to achieve expert status, the aim must still be to direct them towards the development of expertise in the long term” [9]. This is consistent with the proposed theoretical model in Figure 2. Aiming for expertise in students, schools of pharmacy in some nations have implemented a new way of defining and assessing developing pharmacy practice skills; the entrustable professional activities (EPAs) model [34]. A model originally developed in medical education, EPAs are “specific tasks or responsibilities that can be entrusted to a [student] once sufficient, specific competence is reached to allow for unsupervised execution” [35]. Through such a model, students may be able to better-translate competencies to practice in the work place [34].

From an employer’s perspective, the growing evidence for a mismatch between the skills possessed by the workforce and the skills required by employers is well illustrated in many countries, and across health professions [36]. A 2013 report identifying the most important 21st century workforce competencies by analysing the Occupational Information Network data in the US, reported the five most important competencies for most occupations (including health professions) were problem solving, fluid intelligence, team work, achievement/innovation, and communication skills [37], highlighting the diversity and complexities that embody competence. Professional bodies and regulators, supported by professional codes of ethics, also rely on competence standards and frameworks to specify workplace behavioural expectations, to increase visibility of accountability to the public and the rest of the health profession, and to facilitate professional practice and growth [38,39]. And, whilst the rethinking and remodeling of the preceding education models was originally conducted within the context of initial pharmacy education, it is proposed that the evolved competency-based learning and assessment model (Figure 2) is applicable to health care practitioners’ CPD throughout their working lives. In its pursuit, CPD can theoretically be cyclical as depicted in the model, so that with each competency development, purposeful steps to maintain and demonstrate continued competence are made. Ensuring that practitioners attain and maintain competencies as they progress in their professional lives should hold at its ultimate aim improving patient safety and optimizing health outcomes. It is therefore imperative that a practitioner’s CPD journey is well-guided with meaningful feedback, not merely from peers and supervisors but from consumers themselves, whose perceptions and expectations of his/her competence offer yet another dimension to the learning cycle.

## 5. Summary

A conceptual model (Figure 2) was described in this paper seeking to illuminate principles of education models, and can be applied across the diverse contexts of pharmacy and health professions education and in light of the importance of lifelong learning and personal development which are pertinent to professional and clinical performance. It is envisioned that this proposition will continue to be an area for further adoption and research. The new model captured the essence of widely accepted educational paradigms but may have not represented all features, qualities or dimensions of CBE. Simplification of reality whether in academic institutions or workplace environments may have prevailed. Certain assumptions underlying the educational models/theories may have been overlooked. Undoubtedly, a competency-based educational model must not underestimate the implications of factors such as: academic (faculty) capacity and expertise, philosophies and values of education institutions, or multi-disciplinary approaches to learning (e.g., inter-professional collaboration) in practice settings can have on the applicability of such schemes. The model conceptualized should be progressed to integrate feedback from patients/consumers within the learning and assessment paradigms which lends itself to be more useful to the CPD cycle.

In summary, educators/practitioners need to strive for more than just competence and set our “students” (noting that we are all students) up to succeed in an ever-changing environment. Students must be enabled to transfer learning and skills into different paradigms of the ever-changing modern world. Students and practitioners must be instilled with the incentive for lifelong learning. All pharmacy academics, clinical supervisors, students and practitioners may need to take a closer look at how elements of the curriculum/CPD constituents are taught and assessed, learned and transferred to improve our lifelong learning journey.

## References

[1] Hodges B.D., Lingard L. (2012). The Question of Competence: Reconsidering Medical Education in the Twenty-First Century.

[2] Koster A., Schalekamp T., Meijerman I. (2017). Implementation of competency-based pharmacy education (CBPE). Pharmacy.

[3] Austin Z. (2019). Competency and Its Many Meanings. Pharmacy.

[4] Frank J.R., Snell L.S., Cate O.T., Holmboe E.S., Carraccio C., Swing S.R., Harris P., Glasgow N.J., Campbell C., Dath D. (2010). Competency-based medical education: Theory to practice. Med. Teach..

[5] Lockyer J., Bursey F., Richardson D., Frank J.R., Snell L., Campbell C. (2017). Competency-based medical education and continuing professional development: A conceptualization for change. Med. Teach..

[6] Rouse M.J. (2004). Continuing professional development in pharmacy. Am. J. Health Syst. Pharm..

[7] Alsop A. (2000). Continuing Professional Development: A Guide for Therapists.

[8] Alsop A. (2013). Continuing Professional Development in Health and Social Care: Strategies for Lifelong Learning.

[9] McLellan L., Tully M.P., Dornan T. (2012). How could undergraduate education prepare new graduates to be safer prescribers?. Br. J. Clin. Pharmacol..

[10] Miller G.E. (1990). The assessment of clinical skills/competence/performance. Acad. Med..

[11] Biggs J. (2003). Aligning Teaching for Constructing Learning.

[12] Dreyfus H.L., Dreyfus S.E. (2005). Peripheral vision: Expertise in real world contexts. Organ. Stud..

[13] Park J. (2015). Proposal for a modified Dreyfus and Miller model with simplified competency level descriptions for performing self-rated surveys. J. Educ. Eval. Health Prof..

[14] Mehay R., Burns R., Mehay R. (2012). Miller’s Pyramid of Clinical Competence. The Essential Handbook for GP Training and Education.

[15] Persky A.M., Robinson J.D. (2017). Moving from novice to expertise and its implications for instruction. Am. J. Pharm. Educ..

[16] Bajis D., Chaar B., Basheti I.A., Moles R. (2019). Pharmacy students’ medication history taking competency: Simulation and feedback learning intervention. Curr. Pharm. Teach. Learn.

[17] McGaghie W.C. (2015). Mastery learning: It is time for medical education to join the 21st century. Acad. Med..

[18] McGaghie W.C., Issenberg S.B., Cohen E.R., Barsuk J.H., Wayne D.B. (2011). Medical education featuring mastery learning with deliberate practice can lead to better health for individuals and populations. Acad. Med..

[19] Smith L., Krass I., Sainsbury E., Rose G. (2010). Pharmacy students’ approaches to learning in undergraduate and graduate entry programs. Am. J. Pharm. Educ..

[20] Bajis D. (2019). Competency-Based Pharmacy Education and Training in the Eastern Mediterranean Region. Ph.D. Thesis.

[21] (2019). Competence. Merriam-Webster Dictionary [online].

[22] Baumgartner J.L. Continuing Professional Development: What it Means for Student Pharmacists. https://www.pharmacist.com/continuing-professional-development-what-it-means-student-pharmacists.

[23] Accreditation Council for Pharmacy Education (2015). Accreditation Standards and Key Elements for the Professional Program in Pharmacy Leading to the Doctor of Pharmacy Degree-Standards 2016.

[24] Accreditation Council for Pharmacy Education (2019). Policies and Procedures for ACPE Accreditation of Professional Degree Programs.

[25] O’Neill G., McMahon T. (2005). Student-centred learning: What does it mean for students and lecturers. Emerging Issues in the Practice of University Learning and Teaching.

[26] Walshe K., Smith J. (2011). Healthcare Management.

[27] Seligman M. (2018). PERMA and the building blocks of well-being. J. Posit. Psychol..

[28] McLaughlin J.E., Bush A.A., Rodgers P.T., Scott M.A., Zomorodi M., Pinelli N.R., Roth M.T. (2017). Exploring the requisite skills and competencies of pharmacists needed for success in an evolving health care environment. Am. J. Pharm. Educ..

[29] Castillo J.M., Park Y.S., Harris I., Cheung J.J.H., Sood L., Clark M.D., Kulasegaram K., Brydges R., Norman G., Woods N. (2018). A critical narrative review of transfer of basic science knowledge in health professions education. Med. Educ..

[30] Cole S. (2016). Leading the pharamcy workforce for the future. 21st Annual ASHP Conference for Pharmacy Leaders.

[31] Nelson M.H., Fierke K.K., Sucher B.J., Janke K.K. (2015). Including emotional intelligence in pharmacy curricula to help achieve CAPE outcomes. Am. J. Pharm. Educ..

[32] Holmboe E.S., Sherbino J., Long D.M., Swing S.R., Frank J.R., Collaborators I.C. (2010). The role of assessment in competency-based medical education. Med. Teach..

[33] Jungnickel P.W., Kelley K.W., Hammer D.P., Haines S.T., Marlowe K.F. (2009). Addressing competencies for the future in the professional curriculum. Am. J. Pharm. Educ..

[34] Pittenger A.L., Chapman S.A., Frail C.K., Moon J.Y., Undeberg M.R., Orzoff J.H. (2016). Entrustable professional activities for pharmacy practice. Am. J. Pharm. Educ..

[35] Ten Cate O. (2013). Competency-based education, entrustable professional activities, and the power of language. J. Grad. Med. Educ..

[36] Gordon E.E. (2014). A regional focus for solving the skills-jobs mismatch. Surg. Neurol. Int..

[37] Burrus J., Jackson T., Xi N., Steinberg J. (2013). Identifying the Most Important 21st Century Workforce Competencies: An Analysis of the Occupational Information Network (O* NET).

[38] Pharmaceutical Society of Australia National Competency Standards Framework for Pharmacists in Australia 2016. http://advancedpharmacypractice.com.au/download/resources/5316%20Competency%20Standards%20Mapping%20Document_FINAL.pdf.

[39] Pharmacy Council of New Zealand Competence Standards for the Pharmacy Profession. https://enhance2.psnz.org.nz/assets/downloads/group_three/reflection/Standards_2015_FINAL.pdf.

